# Using Mobile Technology to Study Episodes of Alcohol Self-Administration in Daily Life: A Narrative Review

**DOI:** 10.1007/s40429-026-00790-8

**Published:** 2026-09-11

**Authors:** Daniel J. Fridberg, Emily A. Atkinson, Ryan W. Carpenter, Alison M. Haney, Jack T. Waddell

**Affiliations:** 1 Department of Psychiatry and Behavioral Neuroscience, The University of Chicago, Chicago, IL USA; 2 Department of Psychology, University of Toledo, Toledo, OH USA; 3 Department of Psychological Sciences, University of Notre Dame, South Bend, IN USA; 4 Department of Community Health and Health Behavior, University at Buffalo, State University of New York, Buffalo, NY USA; 5 Department of Psychology, Arizona State University, Tempe, AZ USA

**Keywords:** Alcohol use disorder, Ecological momentary assessment (EMA), Alcohol subjective responses, Craving, Drinking topography, Heavy drinking

## Abstract

**Purpose of Review:**

Prior research has identified risk factors for alcohol consumption and development of alcohol use disorder (AUD), but relatively less is known about in-the-moment predictors of risk during real-world drinking events. This narrative review focuses on recent research using event-contingent ecological momentary assessment (EMA) to characterize predictors of alcohol use and drinking-related risk among adults in their natural environments.

**Recent Findings:**

Recent studies using event-contingent EMA, including those employing high resolution EMA (HR-EMA) designs, have highlighted important intra-episode risk factors for heavy drinking including momentary changes in drinking context, alcohol craving, subjective intoxication and alcohol responses, and drinking topography. An emerging body of research suggests that existing event-contingent EMA protocols may benefit further by incorporating new mobile alcohol sensor technologies.

**Summary:**

Supported by recent advances in mobile technologies, event-contingent EMA is a valuable tool for studying how individual differences in-the-moment factors inform alcohol use and drinking-related consequences.

## Introduction

Excessive alcohol use is a major global health burden and leading contributor to disease and death, accounting for approximately 2.6 million deaths and 116 million disability-adjusted life-years lost globally in 2019 [1]. While alcohol use is common around the world [1], heavy drinking is associated with the development and persistence of alcohol-related problems, including alcohol use disorder [AUD; 2].

Given the significant contribution of heavy alcohol use to poor health outcomes, extensive research has examined factors predicting the development and maintenance of hazardous drinking behavior. For instance, demographic factors (e.g., male sex, younger age, student status), family history of heavy drinking and alcohol-related problems, and psychosocial factors (e.g., association with heavy-drinking peers) are established predictors of heavy alcohol consumption and AUD risk [3, 4]. However, comparatively less work has focused on predictors of excessive alcohol use within discrete drinking events (i.e., intra-episode factors). To address this, researchers have employed controlled laboratory alcohol challenge paradigms in which participants receive a fixed dose of alcohol within a prescribed time period [5–7] or “bar lab” approaches with ad libitum drinking offered in a laboratory setting simulating a real-world drinking environment [8–10]. Such laboratory-based approaches, particularly placebo-controlled alcohol challenge designs, are considered the “gold standard” tool for examining intra-episode predictors of drinking as researchers are able to exert a high degree of experimental control over factors such as dose, rate of consumption, and drinking context [11].

Laboratory approaches have provided critical insights into the role of intra-episode factors on drinking behavior and future alcohol-related risk. However, such approaches also have notable limitations, including significant time and monetary resources needed to conduct these studies and limited ecological validity with regard to individuals’ typical drinking environments. For example, data collection for a single participant during an alcohol challenge paradigm may exceed 10 or more hours before they can be discharged home at a safe breath alcohol concentration [BrAC; 12]. Additional limits include the amount of alcohol that can be safely and ethically administered to participants in the lab and prohibitions on administering alcohol to those under the legal drinking age. These restrictions limit the conclusions that may be drawn from laboratory-based work, especially pertaining to factors related to very heavy (i.e., high-intensity) alcohol use events or the generalizability of findings to relevant high-risk groups, such as those below the legal drinking age.

The past 20 years has seen rapid advancement in mobile technologies and high adoption rates of smartphone devices [ e.g., > 97% of American adults aged 18–49 own a smartphone, 13]. These technological breakthroughs have allowed researchers to move “out of the lab and into the wild” by leveraging technology to make it increasingly feasible to capture detailed information on drinking behaviors and related outcomes in near-real-time and in participants’ natural environments. The insights gained from these approaches have expanded our understanding of how individual differences influence drinking behavior and risk in the real world, and may one day inform personalized, targeted treatments for hazardous drinking [14]. At the same time, most of this work has focused on pre-drinking predictors of alcohol initiation [e.g., affect prior to alcohol use; 15] and total quantity consumed, and not on intra-episode factors that may predict, for example, continued drinking or experiencing negative consequences once drinking has begun. Nevertheless, there has been increasing attention paid to the potential value of studying alcohol use episodes as they unfold in the natural environment. Here, we highlight recent work using mobile data collection to explore associations between intra-episode drinking behavior and alcohol-related risk in adults who consume alcohol. In light of rapid advances in technology noted above, and to provide an overview of current trends in the field, we have opted here to focus on recent literature, broadly defined as studies published within the past ~ 5–7 years. We are primarily concerned with reviewing those studies that used mobile technologies, such as smartphones, to capture real-time data on alcohol use and related outcomes from study participants during natural environment drinking events. We searched databases (Google Scholar, PubMed) using keywords (“event-contingent ecological momentary assessment”, “high-resolution ecological momentary assessment”, “alcohol,” “alcohol response”, “craving,” “mobile technology,” “subjective intoxication.”) and used expert consensus by the authors to identify papers and topics for review. Additionally, some preliminary data related to this topic were presented by the authors at a symposium at the annual meeting of the Research Society on Alcohol in 2025 [16], and the scope of this review was further informed by feedback on this symposium from other alcohol researchers. By leveraging mobile technologies, researchers can gain insights into factors contributing to hazardous drinking behavior in the natural environment, which may be difficult, costly, or impossible to observe in a laboratory setting.

## Event-Contingent Ecological Momentary Assessment of Alcohol Drinking Events

Ecological momentary assessment (EMA) refers to real-time sampling of individuals’ self-reported behaviors or experiences in their natural environments; modern EMA methods typically capture these data using participants’ own smartphones. EMA allows for greater ecological validity compared to laboratory-based assessments of alcohol use and minimizes recall bias versus retrospective surveys [17]. EMA protocols commonly used in alcohol research include prompts issued randomly over a period of time (e.g., days or weeks), scheduled prompts issued at the same time(s) daily, or event-contingent prompts issued when a participant reports that he/she is drinking alcohol [17–19] Most studies assess alcohol use in terms of the number of standard drinks (e.g., 1.5 oz liquor, 5 oz wine, 12oz beer) consumed, although the time periods assessed can vary widely, from right at the time of assessment to the previous day [19].

Event-contingent EMA protocols are uniquely suited to assess intra-episode drinking behaviors and outcomes as they do not rely on “catching” participants drinking as with random or scheduled EMA prompts (although these prompts can provide a helpful “safety net” to capture drinking episodes that participants forget to report). Event-contingent EMA can be paired with daily-diary or random prompt EMA to capture additional information on drinking-related events not reported in an event-contingent fashion by the participant [ e.g., drinking-related consequences; 20–23] or to gather other relevant data outside of a drinking event that may be relevant to one’s use behavior during a future alcohol use episode [ e.g., daily changes in mood; 24–26]. While most event-contingent EMA work examining alcohol use has focused on adult samples, this method has demonstrated feasibility in adolescents as well [e.g., 27, 28], supporting the flexibility of the approach across relevant at-risk groups.

High-resolution EMA [HR-EMA; 19] is a type of event-contingent EMA intended to oversample individuals’ experiences during a specific event or behavior of interest. In this type of design, event-contingent reports are used not only to determine that alcohol use has occurred, but also to assess outcomes of interest within the drinking episode. We review some specific applications of this approach below. Earlier HR-EMA studies of alcohol issued participants a device (a pocket computer/personal digital assistant) to record their responses in the field [e.g., 27–31], whereas more recent studies typically use participants’ own smartphones to administer survey prompts [e.g., 18, 20, 32].

Piasecki [19] identified multiple types of event-contingent HR-EMA used to assess alcohol consumption and related outcomes. Although each approach has strengths and limitations, we highlight here the timed follow-up approach. In this method, participants are trained to initiate the assessment protocol when starting or engaging in a target behavior (e.g., alcohol consumption), with subsequent survey prompts issued frequently over a discrete period (e.g., every 30 min for several hours). In the context of an alcohol use event, HR-EMA allows researchers to measure intra-episode changes in drinking behavior and related outcomes (e.g., pace of drinking behavior, mood, and subjective responses to alcohol). Multiple studies have shown that alcohol use can be reliably measured during drinking events using HR-EMA, even during very heavy drinking events and among individuals with severe AUD [20, 21, 24, 30, 32]. The timeframe for assessment is flexible and can be limited to a few hours during a drinking event to minimize participant burden [e.g., 20, 21, 32], or extended dynamically to capture longer drinking events [30, 33].Similarly, the frequency of surveys can be altered depending on the research question. At each survey time point during the HR-EMA period, participants can report their recent alcohol consumption in standard drink units [e.g., 34, 35], or by recording the number, beverage type (e.g., beer, wine, or liquor), and serving size consumed since the prior survey, which is then converted via software to standard drink units [e.g., 32].These data can be used to calculate estimated blood alcohol concentration (eBAC) as an index of the participant’s intoxication over the course of the drinking event [30]. In addition to total alcohol consumed during the episode, additional survey items can capture variables of interest related to drinking context (e.g., location, presence or absence of others), consequences, positive or negative affect, and so on.

## Applications of Event-Contingent EMA to Examine Factors Related to Alcohol Use in the Natural Environment

### Understanding Naturalistic Drinking in Context

One particularly salient area of inquiry in EMA research has been in understanding the role of social context in naturalistic drinking. While laboratory studies can manipulate social context with a high degree of control [e.g., 8, 9] event-contingent EMA is uniquely suited to provide insights into how dynamic shifts in social context during daily life relate to drinking behavior. Daily diary EMA studies have shown that days when individuals drink in social settings, drink with more people also consuming alcohol, or drink in stimulating public settings (e.g., at a bar) are associated with heavier drinking behavior [36–39]. However, event-contingent EMA also allows us to also understand how social context may shift even within a given drinking episode. For instance, using HR-EMA, Waddell and colleagues [18] found that 50% of the variability in social context was due to momentary shifts within the confines of daily drinking episodes (e.g., going from solitary to social drinking). This study and others demonstrated that such within-episode shifts in context are meaningful predictors of subsequent, within-episode drinking behavior. That is, naturalistic use moments within an episode when individuals are in social (versus solitary) contexts are associated with a higher likelihood of subsequent drinking continuation [e.g., 38, 40] as are moments when individuals report being in stimulating environments such as bars/restaurants [41], and moments when individuals report being around intoxicated peers [42]. It has also been shown that drinking with others (versus alone) is associated with increased momentary stimulation and reward [43, 44], which are associated with the development and maintenance of heavy drinking behavior and AUD (see “*Measuring real-time alcohol subjective responses during heavy drinking events*,” below).

### Examining Naturalistic Deviations in Within-Episode Alcohol Craving

Craving remains an important yet understudied process in the addiction sciences. Many theories of alcohol craving focus on how craving leads to alcohol use, and vice versa, over time. The Cognitive Processing Model [45] suggests that, as AUD develops, alcohol use becomes an increasingly automatic and habitual response to triggers, while craving remains a cognitive and conscious process that leads to drinking (or not) only sometimes. As such, recent event-contingent EMA studies have begun to outline the processes through which craving manifests and continues during naturalistic use episodes, as well as the antecedents and outcomes of this relationship. Above and beyond the social contextual factors and subjective responses to alcohol mentioned above, craving has been identified as a strong and highly salient predictor of continued, in-the-moment drinking [e.g., 18, 41]. Waddell and colleagues [46] found that relations between naturalistic craving and drinking behavior are cyclic, wherein early-episode craving potentiates the quantity of consumption thereafter, which then augments craving and further consumption. Thus, from a motivational perspective, within-episode shifts in craving, particularly early in an episode, may be ideal times to intervene before drinking continues and, over time, cements greater consumption that predisposes risk for acute alcohol-related harms.

Several event-contingent EMA studies have also outlined factors that are associated with deviations in craving assessed during acute use episodes, including perceptions of peer intoxication [24], subjective affective responses to alcohol [47] presence of and reactivity to alcohol cues [48] and simultaneous use of alcohol and other drugs [e.g., 38, 40]. Across studies, event-contingent EMA methods have demonstrated the importance of measuring within-episode deviations in craving/wanting and have outlined both precipitants and downstream outcomes of such deviations as they naturally occur.

### Understanding Discrepancies Between Objective Measures of Alcohol Consumption and Subjective Intoxication

Event-contingent EMA approaches are particularly well-suited to capture variability between objective indices of consumption (i.e., BAC; eBAC) and individuals’ subjective experiences of intoxication across time and drinking context. Notably, recent advances in computational modeling and mobile technology have facilitated exploration of how discrepancies between subjective and objective intoxication may be associated with alcohol-related consequences in daily life. For example, though subjective and objective intoxication indicators are moderately associated (*β*s = 0.5–0.6; measured via eBAC and transdermal alcohol concentration), these associations vary between individuals and across days [34]. Further, episode peak subjective intoxication has been shown to predict consequences above peak eBAC and peak TAC [34]. This suggests that within-person measurement of subjective intoxication in a daily life context offers unique information when predicting consequences above and beyond alcohol concentration.

Recent exploratory work using machine learning showed that a wide range of factors influence the association between subjective and objective intoxication in the moment, including drinking context, craving, demand, affect, location, and the presence of companions, with time spent drinking and time of day as particularly important indicators [49]. In this sample, subjective intoxication was assessed by asking participants, “*How intoxicated do you feel right now?*” on a Likert scale from 1 (not at all) to 10 (more intoxicated than you’ve ever been). Overall, predictive accuracy of subjective intoxication by machine learning models in this random-prompt EMA sample ranged from 66.7% (decision tree model) to 75.0% (gradient boosted trees model). Further analysis indicated that the set or combination of factors that were most predictive of momentary subjective intoxication varied across individuals [49]. This work, while preliminary, highlights how machine learning approaches based on event-contingent EMA data may be used to model the interrelated and context-dependent factors that can influence an individual’s subjective intoxication and risk of consequences in a particular moment. Given the complexity and variability in these associations, recent advances in computational modeling techniques in combination with event-contingent EMA of alcohol use episodes may allow for novel, individualized approaches to understanding the subjective effects of alcohol and risk for alcohol-related consequences [50].

Relatedly, event-contingent EMA may be especially helpful when studying the phenomenon of acute tolerance to alcohol in naturalistic settings. Acute tolerance refers to a decrease in alcohol’s subjective effects and behavioral impairment within a drinking episode over time, despite active and significant BAC [51, 52]. Acute tolerance has been implicated in the etiology of substance use disorders and may serve as a behavioral risk marker for problematic drinking and chronic tolerance [53, 54]. In the laboratory, acute tolerance can be measured using “steady-state” methods that employ intravenous administration to maintain a constant BAC such that observed reductions in alcohol’s effect can be attributed to a time effect. Alternately, the “Mellanby method” tests for a significant decrease in alcohol’s effect from the ascending to descending limb of the BAC curve at matched BACs. Though well-documented in human laboratory studies [55, 56] and non-human animal models [57–59], acute tolerance has yet to be well-characterized in the natural environment. One recent study reported that young adult heavy drinkers showed evidence of initial increased stimulation to alcohol followed by a “plateau” in ratings of that effect during a natural-environment drinking event, with this effect most pronounced in those who drank alcohol the fastest [60]. The authors speculated that this could reflect individuals drinking faster to overcome tolerance to alcohol’s stimulating or rewarding effects as the episode progresses but noted that the study was not designed to test this hypothesis directly. The advantages of event-related EMA for capturing simultaneous data on time, eBAC, and contextual factors associated with real-world drinking that cannot be easily captured within a laboratory setting make it well-suited for studying acute tolerance in the natural environment, and a promising avenue for future research.

### Measuring Real-Time Alcohol Subjective Responses During Heavy Drinking Events

The subjective effects of alcohol vary across individuals, may be classified in multiple ways beyond overall intoxication, and have been identified as an endophenotype for AUD risk [61]. Decades of laboratory work has supported the role of individual differences in responses to alcohol’s stimulating, sedating, and rewarding effects in risk for heavy drinking and the development of AUD. In these studies, participants received a dose of alcohol (e.g., 0.8 g/kg) either orally [4, 5, 7, 62] or intravenously [63–65] and were asked to rate how they feel at various points along the BAC curve. These studies found that increased sensitivity to alcohol’s stimulating and rewarding properties, along with decreased sensitivity to its sedating effects, is associated with increased risk of hazardous drinking behavior and development and maintenance of AUD. These associations may be more pronounced at high (e.g., 4–5 drink equivalent, in line with a binge drinking event) versus low (e.g., 2–3 drink equivalent) alcohol doses [5].

Recent work implemented smartphone-delivered HR-EMA to extend these results beyond the laboratory setting and into the natural environment. Data from these studies showed that, during typical real-world heavy drinking events, participants reported increased stimulation, hedonic reward (i.e., alcohol liking), and motivation reward (i.e., wanting more alcohol) as eBAC increased [20, 21, 24] In contrast to findings from laboratory studies [e.g., 64], subjective sedation remained generally low during the drinking events, increasing only modestly over the 3-hour HR-EMA period. Importantly, alcohol-related outcomes measured via HR-EMA differed meaningfully across drinking risk groups: relative to lighter alcohol users (i.e., those who consumed 1–9 drinks/week and endorsed rare binge drinking of ≤ 1 occasion per month), risky alcohol users (i.e., those who reported at least weekly binge drinking with no-to-mild AUD, and those with moderate-severe AUD) reported greater alcohol-related stimulation, liking, and wanting more during the 3-hour HR-EMA period, with no differences between groups on reported alcohol-related sedation [21]. These group differences were present even when accounting for differences in intoxication during the episode, consistent with notions that elevated reward and stimulation in response to alcohol may be an endophenotype conferring alcohol-related risk [5, 61].

Smartphone-based HR-EMA can inform our understanding of how alcohol use and subjective responses are related to drinking-related risk in diverse clinical groups. Recently, King and colleagues [24] used HR-EMA to examine the role of positive (i.e., rewarding effects) versus negative (i.e., relief of negative affect) reinforcement in drinking behavior among people with and without AUD and/or comorbid depressive disorder. Results showed that participants with AUD, regardless of comorbid depression status, reported positive stimulant and rewarding effects of alcohol during a drinking event, while negative affect decreased slightly over the course of the episode for all participants regardless of AUD or depression status. These results suggest that alcohol-related reward persists in at-risk individuals even in the setting of depression and challenges the notion that over time heavy drinking becomes a behavior that is motivated primarily by negative, rather than positive, reinforcement [66, 67]. Individuals who use alcohol together with other substances (i.e., polysubstance use) are another group that has received attention. While the HR-EMA work above asked participants to refrain from other substance use during the alcohol use assessments, several groups have modeled the effect of polysubstance use, primarily cannabis and nicotine use, on alcohol response and related outcomes. For example, two studies using event-contingent EMA in young adults found that simultaneous alcohol and cannabis use is associated with increased subjective responses to alcohol, particularly stimulation, and that simultaneous alcohol and cannabis use predicted continued drinking during event-contingent EMAs via augmented alcohol craving [38, 41, 68]. Other studies reported similar effects of using cigarettes and/or nicotine products while drinking, which may also augment acute positive reinforcement from alcohol [40, 69]. Thus, advances in modeling of event-contingent EMA and HR-EMA drinking episodes can either constrain against the use of other substances or model such variability as indicated by the research question and population of interest.

It is important to note that accurate assessment of alcohol use in EMA requires participant compliance with completing survey prompts during drinking events and, depending on the research question, non-drinking occasions as well [e.g., morning report surveys completed the day after a drinking episode, 70]. This may be particularly relevant when capturing heavy drinking episodes characterized by higher levels of impairment, though some work has demonstrated that drinking quantity may not influence event-contingent compliance rates [71]. As overall compliance for a given EMA protocol may differ from compliance during drinking episodes per se, researchers should calculate and report these compliance rates separately. Mobile sensors (e.g., Bluetooth breathalyzers, see below) may also be useful to objectively identify drinking events and examine compliance with morning reports to determine if there are significant differences in response likelihood based on the amount of alcohol consumed (based on peak eBAC or BrAC) the prior evening [72].

### Assessing Drinking Topography in Daily Life

Episodes of alcohol use in the natural environment are complex and dynamic events, over which alcohol consumption ebbs and flows. While total quantity of alcohol consumed is an important aspect of use, it is only modestly associated with AUD [73, 74] and the experience of alcohol-related negative consequences [75]. Using event-contingent EMA methods, researchers can examine the topography of daily-life drinking (i.e., examining not only *how much*, but *in what way* people drink) to gain a fuller understanding of how individual drinking episodes contribute to the development of alcohol-related problems and, in turn, how the development of AUD may impact how people consume alcohol. Although examining drinking topography is feasible in the lab in the context of ad libitum administration protocols [e.g., 10], the ability to examine drinking patterns is constrained by limitations in terms of time and the amount of alcohol that can be given to participants.

The rate or speed of alcohol consumption may be a particularly important component of drinking episodes. People can drink the same quantity of alcohol at different speeds (e.g., taking a shot vs. sipping a beer) and drinking faster causes BAC to rise at a faster rate, which contributes to more time spent overall at elevated levels of intoxication. Several EMA studies have found that people experiencing or at risk of experiencing alcohol-related problems drink at elevated rates. This work modeled rate of consumption as the change in eBAC relative to the start of the drinking episode, testing for group differences at each scheduled follow-up. Carpenter et al. [76] found that young adults with more symptoms of AUD drank faster than people with fewer symptoms, McNamara et al. [77] found that people with higher trait impulsivity drink faster than people with lower impulsivity, and Carpenter et al. [78] found that people with borderline personality disorder [a psychiatric illness highly comorbid with AUD; 77] drank faster than community comparisons. Critically, in all studies, there were no differences in the amount of alcohol consumed despite differences in rate of consumption.

Additional research in this area has used event-contingent EMA to characterize relevant outcomes related to individual differences in alcohol consumption patterns. It may be the case, for instance, that people drink at elevated rates to maximize the positive, hedonic effects of alcohol (i.e., to get the “most bang for their buck” from their alcohol use). Evidence from event-contingent and HR-EMA work supports this notion. These studies showed that drinking fast is associated with greater subjective stimulation, reward, and positive affect, as well as reduced negative affect [22, 60, 78]. Unfortunately, EMA research suggests that rapid alcohol use may come at a significant price, as elevated rate of consumption has been linked to greater risk of experiencing blackouts and other negative consequences [22, 60, 79]. Notably, Carpenter & Merrill [22] found that rate of consumption was uniquely associated with the likelihood of experiencing negative consequences independent of the effect of the amount of alcohol consumed (which was also associated with negative consequences). In sum, these data suggest that considering drinking topography, in addition to total amount consumed, may be important to fully understand alcohol use, its antecedents and consequences, and how alcohol-related problems develop over time. Event-contingent EMA approaches are well-suited for studying this important aspect of alcohol use in individuals’ natural environments. Examining associations between specific aspects of drinking topography, such as drinking speed, and participant response patterns could also aid researchers in identifying ways to enhance compliance with EMA of real-world alcohol use (e.g., by optimizing question formatting or prompt scheduling to facilitate data collection even during rapid-drinking events).

## Future Directions: Integrating Event-Contingent EMA with Ambulatory Mobile Technologies

Future work implementing event-contingent EMA of naturalistic drinking events should focus on improving the accuracy and reliability of this approach in diverse drinker subgroups. One particularly promising area concerns integrating additional devices that can passively (e.g., transdermal alcohol biosensors) or actively (e.g., portable breathalyzers) measure physiological indices of alcohol consumption. An advantage of these devices is that they can provide a physiologically-based measure of real-world alcohol consumption to complement self-report during EMA. As a result, they rely significantly less upon participants to provide accurate and timely information about their alcohol use. While a full review of the literature integrating EMA and ambulatory alcohol sensor technology is beyond the scope of the present article, we provide an overview here of key considerations and note that recent reviews have covered this topic in more detail [14, 80–82].

Transdermal alcohol sensors, worn around the wrist or ankle, record transdermal alcohol concentration (TAC) by continuously and passively monitoring alcohol excreted through sweat [83, 84]. Work in this area has focused on estimating BrAC/BAC from TAC [85–87] and examining associations between specific features of the TAC signal (e.g., rise rate, peak, area under the curve, and fall rate) and alcohol use and drinking-related consequences [34, 84, 88–91].

Portable breathalyzers, small enough to be easily carried and capable of connecting wirelessly with other devices, can be used to accurately sample breath alcohol concentration (BrAC) across a drinking episode [92, 93]. Recent work has examined the association between BrAC recorded in the field via portable breathalyzers and self-reported alcohol use and related constructs. Haney et al. [92] had participants complete both a laboratory alcohol challenge paradigm and an EMA protocol and compared associations between mood and BrAC across contexts. Greater momentary excitement was associated with a higher BrAC in daily life, but a lower BrAC in the laboratory, even when accounting for limb effects and differences in BrAC range between contexts. This highlights how meaningful, contextual differences between research contexts (e.g., environment, companions) may influence associations between subjective states and intoxication, even when using the same assessment approaches and the same participants. Motschman et al. [94] found that greater self-reported behavioral economic demand for alcohol was associated with both greater self-reported alcohol use and BrAC.

Despite the significant potential of ambulatory devices for assessing daily life alcohol use, it is important to keep in mind that they have limitations. First, while these devices reduce the risk of participant error, they still rely on participants to keep them charged, wear and use them correctly, and avoid losing or breaking them. Related to this, these devices also increase the burden placed upon participants and not all potential participants may be willing to wear a device or blow into a breathalyzer in public [93]. Second, there are multiple versions of these devices available for purchase and the number of options is likely to only increase. It is important for researchers to be aware that these devices are not all equally reliable or valid and not all available devices have been rigorously tested by researchers e.g. [90]. Transdermal sensors have also been found to be better at detecting elevated alcohol use than light use [95–97]. TAC also lags behind BAC. Although this lag has shortened with the current generation of sensors [98], this lag, which varies across person and episode, makes it more difficult to use for assessing events in the moment as they occur [99]. Portable breathalyzers, in turn, can be impacted by residual mouth alcohol, requiring participants to pause their drinking to ensure an accurate reading.

## Limitations of EMA Self-Report to Assess Naturalistic Alcohol Use Events

While EMA of real-world drinking episodes has important advantages over laboratory-based approaches in terms of ecological validity and cost and resources to administer, there are important limitations to consider. Perhaps the most significant is that participants may misreport the amount of alcohol they consume. Although participants can be trained to report in terms of standard drinks (e.g., 1.5 oz liquor, 5 oz wine, 12 oz beer), alcoholic beverages in the natural environment do not always neatly match these categories, either in terms of size (e.g., a 12 oz bottle vs. a pint vs. a Solo cup) or percent alcohol (e.g., a domestic light beer (~ 4% abv) vs. a craft bourbon-barrel aged imperial stout (≥ 10% abv)). In particular, accurate estimation of standard drinks can be highly challenging with mixed drinks as they may contain multiple “shots” of spirits exceeding a single standard drink (1.5 oz), with contents differing by venue. Accurately estimating drink size during real-world drinking events may be especially difficult when distracted by friends or the environment, and when experiencing the cognitive impairments associated with alcohol use. It may be possible to mitigate this limitation by asking participants to report the type, serving size, and number of drinks consumed since the last survey prompt to estimate the number of standard drinks consumed [32]. As noted above, EMA studies of natural environment alcohol consumption are often interested in measuring changes in relevant constructs (e.g., mood, alcohol subjective responses) in response to alcohol’s pharmacological effects. This raises questions about the accuracy of reporting during intoxication, as well as whether the observed effects may be attributed to alcohol’s effects versus other individual or environmental factors. In the laboratory, this limitation can be mitigated by implementing a blinded placebo-controlled alcohol challenge design [11, 61]. An analogous design in the natural environment is more challenging as individuals in that setting typically are not blind to beverage type. To partially mitigate this issue, we (DJF) ask participants in our EMA studies to record their experiences during a typical non-alcohol drinking event for comparison with those same outcomes recorded during a drinking episode [21, 24]. The assessment period of the non-alcohol drinking event is the same as that of the alcohol-drinking event (to account for the passage of time), and participants are encouraged to match other characteristics of both events (e.g., time of day, location) to the extent possible. While not a perfect analog for a laboratory placebo control, this approach allows investigators to account for individual difference factors unrelated to alcohol consumption in the data, and may help isolate effects of interest specific to alcohol consumption. To reduce the possible impact of cognitive impairment, researchers should also ensure that they are following consensus guidelines for optimal EMA item selection and design, which include using clear and straightforward language (e.g., avoiding ambiguous or reverse-scored items) in easily readable font [100].

A related limitation is that participants may refrain from reporting any alcohol use at all if they do not wish to receive subsequent EMA prompts during an event, or if they believe that completing the surveys will be burdensome. Some ways to address this include providing participants with a clear rationale for the study purpose and importance of reporting alcohol use and completing follow-ups, allowing participants to dismiss/silence prompts when retiring for the evening [30] restricting assessments to only certain types of drinking events e.g., typical heavy drinking episodes; [21, 65], and limiting the total duration of the each episode assessment [32]. Missing data due to unintentional noncompliance is also a concern in EMA of alcohol use, as intoxication or the nature of the drinking environment (e.g., a loud party) may contribute to missed survey prompts. Experimenters can mitigate the risk of missing data by training participants ahead of time on how to use the EMA interface and the nature of the questions that will be asked, incorporating a clear, user-friendly interface (e.g., large buttons, brief phrasing for questions, easy-to-follow directions), deploying reminder prompts if a survey is not completed within a given time, and monetary incentives for completing above a given percentage of surveys [e.g., 18, 32].

Current multilevel modeling techniques, which account for the nested and unevenly spaced nature of EMA data (e.g., moments nested within individuals) can accommodate missing data. However, it is important to consider whether missingness is at random or, instead, related to systematic factors like degree of intoxication and time of day or elapsed time since drinking started. It is particularly important to be mindful of how such systematic factors could potentially bias findings (e.g., by reducing the number of observations in the data at elevated intoxication). When planning analyses, it is also important to consider the frequency of both the number of assessments within drinking episodes and the number of drinking episodes across people. Although EMA, in general, makes it possible to collect a large number of within-person observations, leading to increased power, analyses focused on drinking episodes will likely be constrained to only those survey prompts issued during drinking events. If participants drink infrequently during the EMA protocol and/or they complete few survey prompts during episodes (either due to study design or low compliance), there may be insufficient power to examine effects of interest. For this reason, it may be beneficial to recruit participants who drink regularly (e.g., weekly or more), employ sufficiently long EMA protocols to capture multiple drinking episodes, and include a greater number of event-contingent survey prompts. Of course, the potential benefits of a longer EMA protocol or more survey prompts must be balanced against the increased costs in terms of participant compensation and burden.

Last, as many real-world drinking events take place in the evening, it may be difficult or impossible to consistently capture event-contingent EMA when BAC is declining, as participants may be asleep during that time [19]. Note that this may mean that some people have limited experience with, e.g., subjective experiences encountered during the descending limb of the BAC curve in their daily lives. That said, one means of potentially capturing experiences while BAC is declining could be to target “day-drinking” events (e.g., sporting events) where participants may be more likely to be awake during all or most of the BAC curve.

These limitations speak to the need for careful attention to detail in study design, participant training, and selecting research questions that maximize the advantages of event-contingent EMA to examine alcohol use episodes. It is also important to note that the available evidence, though somewhat limited, suggests that self-reported alcohol use is generally reliable and accurate via EMA [96, 101–104]. Given that all methods of assessment will have limitations, the best approach to accurately assessing alcohol use may not be adversarial (i.e., identifying the best method) but collaborative (i.e., integrating information across methods). With different methods (e.g., self-report, TAC, BrAC) potentially subject to different sources of measurement error, combining them (e.g., in a latent variable approach) could provide a clearer picture of daily-life drinking episodes than any method alone. Future work should explore this possibility.

## Conclusions

EMA, supported by the widespread proliferation and use of smartphones, provides researchers with new opportunities to examine critical factors related to heavy alcohol use and the development and maintenance of AUD. In this paper, we highlighted recent applications of event-contingent EMA to examine associations between dynamic intra-episode factors (drinking context, alcohol craving, subjective effects and acute tolerance, and drinking topography) and outcomes related to drinking-related consequences and risk. It is important to note, however, that this approach can be tailored as needed to study a wide range of alcohol-related outcomes beyond those reviewed here (e.g., alcohol-related risk taking, drinking-related consequences, hangover). Event-contingent EMA, and HR-EMA in particular, is particularly well-suited to study factors related to hazardous drinking outside of the laboratory and in diverse drinker groups in real-time and across environmental and social contexts. It is also important to note that a great deal of work cited here was based upon observations originally recorded in the laboratory setting, and both laboratory and EMA paradigms feature strengths and weaknesses. Similarly, while we highlight event-contingent approaches here, other EMA methods (e.g., daily diary or random prompt) may be more appropriate depending on the research question asked. We also highlighted limitations of event-contingent EMA methods to study alcohol use, including participant reporting errors (e.g., in terms of alcohol consumed), and missing or inaccurate reports caused by alcohol intoxication. Newer technologies, such as alcohol biosensors or portable breathalyzer devices, may possibly be helpful to offset some of these concerns, and research on the best ways to integrate these devices with EMA methods continues. There is also significant potential in incorporating other ambulatory devices capable of collecting geolocation, psychophysiological indices, and other objective measures. In sum, event-contingent EMA is a flexible approach that may be used to study multiple aspects of real-world alcohol consumption. As mobile technology continues to advance, we expect that this approach will continue to prove highly useful in furthering our understanding of key factors related to alcohol-related risk, which could in turn inform novel interventions targeting hazardous drinking.

## Data Availability

No datasets were generated or analysed during the current study.
